# Associations between childhood trauma, depression, anxiety disorders and early arthritis presence

**DOI:** 10.3389/fmed.2025.1582075

**Published:** 2025-08-05

**Authors:** Ana Konjikusic, Sarah Ohrndorf, Tanja Braun, Vivien Kluckow, Vera Höhne-Zimmer, Gabriela Schmittat, Christine Heim, Ralf Uebelhack, Kirsten Minden, Jacqueline Detert, Gerd-Rüdiger Burmester, Desiree Schaumburg

**Affiliations:** ^1^Department of Rheumatology and Clinical Immunology, Charité - Universitätsmedizin Berlin, Freie Universität Berlin and Humboldt Universität zu Berlin, Berlin, Charitéplatz, Germany; ^2^Charité - Universitätsmedizin Berlin, Freie Universität Berlin and Humboldt Universität zu Berlin, Institute of Medical Psychology, Berlin, Charitéplatz, Germany; ^3^NeuroCure Cluster of Excellence, Charité - Universitätsmedizin Berlin, Universität Berlin and Humboldt Universität zu Berlin, Berlin, Charitéplatz, Germany; ^4^Department of Psychiatry and Psychotherapy, Charité - Universitätsmedizin Berlin, Freie Universität Berlin and Humboldt Universität zu Berlin, Berlin, Charitéplatz, Germany; ^5^Deutsches Rheuma-Forschungszentrum Berlin, Epidemiology Unit, Charité - Universitätsmedizin Berlin, Freie Universität Berlin and Humboldt Universität zu Berlin, Berlin, Charitéplatz, Germany; ^6^Department of Pediatric Respiratory Medicine, Immunology and Critical Care Medicine, Charité - Universitätsmedizin Berlin, Freie Universität Berlin and Humboldt Universität zu Berlin, Berlin, Charitéplatz, Germany; ^7^Rheumatologisch-immunologische Praxis Templin, Templin, Germany

**Keywords:** early arthritis (EA), depression, childhood trauma, anxiety, PTSD - posttraumatic stress disorder

## Abstract

**Objective:**

To compare the prevalence of childhood trauma, depression, and anxiety disorders between patients with early arthritis (EA) and a control group (CG). We further aimed to explore the influence of these variables on EA diagnosis.

**Methods and materials:**

This monocentric study included 60 prospectively recruited EA patients with at least one inflammatory joint with a symptom duration of 1–12 months. The CG consisted of 60 individuals with no clinical signs of arthritis. The participants underwent a semi-structured interview screening for psychiatric disorders and completed standardized questionnaires, including the Hospital Anxiety and Depression Scale (HADS-A, HADS-D), the Childhood Trauma Questionnaire (CTQ), and the Posttraumatic Diagnostic Scale (PDS). For statistical analysis, we used SPPS© χ^2^ test, T-test, Mann–Whitney U-test, and binominal regression analysis.

**Results:**

Compared to the CG, patients with EA had significantly higher rates of depression in the interview (41.7% vs. 16.7%; *p* = 0.03) and PTSD (13.3% vs. 3.3%; *p* = 0.048), and significantly higher HADS-D and CTQ childhood sexual abuse mean scores (HADS-D: 5.40 ± 4.80 vs. 3.60 ± 3.30; *p* = 0.047; CTQ sexual abuse: 5.91 ± 2.68 vs. 5.15 ± 1.02; *p* = 0.042). Binomial regression analysis revealed higher odds ratios for EA for CTQ emotional neglect (*p* = 0.048, OR = 1.12), CTQ sexual abuse (*p* = 0.040, OR = 1.4), HADS-D (*p* = 0.025, OR = 1.12), and lifetime depression (*p* = 0.040, OR = 4.00).

**Conclusion:**

The high rates of depression and PTSD in EA emphasize a potential link between psychiatric disorders and arthritis. The presence of EA might be associated with present and lifetime depressive symptoms, childhood sexual abuse and emotional neglect.

## 1 Introduction

### 1.1 Background

Rheumatic diseases can present with a wide variety of clinical symptoms. They are mostly chronic, incurable and cause pain, leading to disability and ultimately functional impairment (1, 2). Patients with chronic arthritis often exhibit impaired psychological well-being (3). Overlap between autoimmune and psychiatric diseases has long been known (4). Many studies have reported a high prevalence of symptoms of depression and anxiety among patients with established arthritis (5, 6). Only recently has a growing amount of data shown increased psychological distress in patients in the early stages of arthritis (7, 8).

It is assumed that there is a bidirectional relationship between stress and inflammation (9), as stress and trauma can trigger long-lasting effects and enhance arthritis (10). Depression is a possible risk factor for inflammatory joint diseases (11). Depression is associated with increased levels of proinflammatory cytokines, such as tumor necrosis factor-*α* (TNF-α), which also plays a key role in the pathophysiology of rheumatic disorders (12). Psychological well-being affects the outcome of rheumatic diseases, as the coexistence of both depression and anxiety is associated with a 50% decrease in therapy response (13).

Moreover, several authors have suggested a link between childhood trauma and a possible increase in proinflammatory cytokines, such as interleukin 6 (IL-6), TNF-*α*, and C-reactive protein (CRP), which are known to be associated with autoimmune diseases and chronic inflammatory diseases (14, 15). One mechanism that may promote inflammation after stress in childhood involves long-term effects on the regulation and reactivity of the hypothalamic–pituitary–adrenal (HPA) axis, which is associated with altered cortisol production and resistance of glucocorticoid receptors in target cells, such as immune cells, ultimately promoting inflammation in joints (16). Further studies suggest that childhood trauma is more prevalent in patients with arthritis (10, 17) and might have long-term effects on inflammatory and autoimmune diseases, leading to more frequent hospitalizations in adulthood (18). Another considerable psychiatric risk factor for RA might be PTSD (19). Veterans who had been deployed to Afghanistan and Iraq were twice as likely to suffer from autoimmune diseases when previously diagnosed with PTSD (20). PTSD severity is also associated with increased levels of proinflammatory cytokines such as TNF-*α* (21). However, how different psychiatric factors and traumatic events influence the occurrence of different forms of arthritis has yet to be fully elucidated.

This study aims to assess the prevalence of psychiatric disorders, such as depression and anxiety disorders, childhood trauma, and PTSD, in a clinical sample of early arthritis (EA) patients versus controls. We predominantly included treatment-naive patients in the early stages of rheumatic disease to minimize the influence of pain chronification, immobility, and the adverse effects of anti-inflammatory medication on psychological well-being. Furthermore, we aimed to determine whether these psychiatric conditions are associated with the presence of EA. Finally, we explored the link between depression and laboratory and clinical features in EA patients.

## 2 Methods and materials

### 2.1 General information

This monocentric study comprised 120 participants. This study represents part of an analysis of the cross-sectional data of the PSYRA study, which was designed to assess psychological comorbidities in the context of rheumatic and musculoskeletal diseases in an early arthritis clinic. PSYRA is a monocentric longitudinal study with follow-ups of patients with EA and patients with non-inflammatory musculoskeletal diseases. A case number of approximately 60 patients with a statistical test power of 80% (corresponding to a *β*-error probability of 20%) and an alpha error of 0.5 per group was calculated on the basis of similar studies (7). The study included 60 patients with EA and 60 age- and gender-matched controls with no evidence of rheumatic disease to date. Study participants were prospectively recruited between 2015 and 2021. The inclusion criteria for all the participants were an age ≥ 18 years, the ability to read and comprehend the study-related documents, the capacity to consent and not to be involved in any legal issue/procedure. All the participants signed the informed consent document. Ethical approval was obtained from the local ethics committee of Charité - Universitätsmedizin Berlin (ethical approval number EA1/063/15). There were additional inclusion criteria for the different assigned groups, which are listed in the following two paragraphs.

The EA group comprised patients with early arthritis as a main symptom of immune-mediated rheumatic diseases. These patients attended the early arthritis clinic in the outpatient and inpatient units of the Department of Rheumatology of the Charité - Universitätsmedizin in Berlin. EA was defined, according to EULAR, as the presence of at least one inflammatory joint (presence of clinical synovitis) between 4 weeks and 12 months (22). An experienced rheumatologist verified the presence of EA by clinical examination. Furthermore, acute phase reactants as C-reactive protein (CRP), erythrocyte sedimentation rate (ESR), and antibodies (especially rheumatoid factors/antibodies against citrullinated peptides) were evaluated. In case a general practitioner suspected an inflammatory joint disease, the patient was sent to an early arthritis clinic to validate the diagnosis. After the first consultation at an early arthritis clinic, patients were asked if they wanted to participate in the study. Several patients who agreed to participate in the study were at the clinic with a suspected but later excluded rheumatic condition. These patients were allocated to the furtherly explained non-inflammatory arthralgia group.

The control group (CG) included 60 individuals without signs of inflammatory joint disease manifestation. This group included two subgroups, differentiating participants with NIA from the outpatient clinic and healthy controls without any joint pain or known joint condition, which was subsequently described as no arthralgia (NA). The participants with NIA initially presented at the early arthritis clinic due to arthralgia. Due to normal clinical and laboratory findings with no evidence of inflammation or antibodies, an inflammatory joint disease was excluded by a rheumatologist, and the participant was classified as NIA. The office of a general practitioner was used to recruite the NA group, in case patients had no known joint conditions.

### 2.2 Materials

The EA patients who presented to the early arthritis clinic underwent standard clinical examination and blood check-ups. Documented diagnoses and clinical and laboratory data were used for study purposes if the patients agreed. For EA patients, we analyzed inflammatory parameters in the blood, CRP, and ESR. As relevant elevation was determined CRP > 5 mg/L and ESR > 30 mm/h. Patients underwent standard rheumatological antibody diagnostics with standardized cut-off values. We documented rheumatoid factor IgG/IgM (RF-IgG/IgM) and anti-citrullinated circulating peptide (anti-CCP) levels. The levels of vitamin D were also documented if available. We distinguished between moderate (< 50 ng/mL) and severe vitamin D insufficiency (< 30 ng/mL). The clinical parameter DAS28CRP refers to our RA subgroup. Moreover, we documented a visual analog scale for pain (VAS) score and morning stiffness. Furthermore, we documented whether EA/NIA patients at the moment of participation had already received anti-inflammatory therapy, such as glucocorticoids or conventional synthetic disease-modifying antirheumatic drugs (csDMARD). The NA participants were not referred to the inpatient or outpatient Department of Rheumatology and Clinical Immunology; therefore, their laboratory data were not recorded. Additionally, we assessed common cardiovascular and metabolic risk factors in all participants.

*A semistructured interview*is a standardized research instrument for identifying psychological comorbidities via a semistructured interview according to the Diagnostic and Statistical Manual of Mental Disorders (DSM-V) criteria (23). We focused on assessing psychiatric diagnoses such as current and lifetime depression, anxiety, phobia, PTSD, and childhood adversities. For this purpose, two physicians/researchers from the rheumatology department were instructed by a specialist in psychiatry to conduct this interview and make the diagnosis. For the purpose of statistical analysis, psychiatric diagnoses were clustered into groups: mild, moderate, severe, and recurrent depression were classified as depressive disorders, and generalized anxiety, phobia, and PTSD were classified as anxiety disorders. If more than one outcome was diagnosed during the interview, we documented the presence of a second outcome as well.

Hospital Anxiety and Depression Scale (HADS) is a widely used reliable questionnaire divided into 2 subscales, assessing depressive symptoms and anxious symptoms (24). Each subscale comprises 7 items, each ranging from 0 to 3. The minimum subscale score is 0, and the maximal score is 21. A score > 7 is the cut-off for mild clinical depression/anxiety with a higher sensitivity and a score > 10 is a research-relevant depression/anxiety which presents with higher specificity (25). We presented the outcomes for both scores.

Childhood Trauma Questionnaire (CTQ) is a 28-item self-report questionnaire assessing several childhood trauma dimensions. These include 5 subscales: Emotional Abuse (EA), Physical Abuse (PA), Sexual Abuse (SA), Physical Neglect (PN), and Emotional Neglect (EN). These 5 subscales each comprise 5 items. The participants evaluated the items on a five-point Likert scale, and the score determined for each subscale ranged between 5 and 25. The remaining 3 items fall into an additional subscale, which measures childhood trauma trivialized by the study participants. This score is not included in the total CTQ score. This questionnaire is a reliable and widely used instrument with very good internal consistency and reliability (26–28). Each subscale has a score that denotes severe childhood trauma. The threshold values for the subscales were EA ≥ 13, PA ≥ 10, PN ≥ 10, SA ≥ 8, and EN ≥ 15 (26).

Posttraumatic Diagnostic Scale (PDS) is an instrument designed for screening and diagnosing posttraumatic stress disorder (PTSD). This questionnaire is concordant with the DSM-IV (29) and includes six criteria for PTSD (A to F). For the PTSD diagnosis, all criteria have to be met and verified via an interview (30). This self-administered questionnaire has 49 items and is divided into 4 sections. In the first and second sections, there is a checklist of 12 potential traumatic experiences in which patients are supposed to check the events they experienced or witnessed and express how they felt during the experience. The third section, Symptom Severity Score, refers to criteria B to D, covering symptoms of re-experiencing/intrusion, avoidance, and arousal (31, 32). The last section presents the loss of function in various areas of functioning. Foa et al. estimated that the coefficient of reliability was Cronbach’s alpha = 0.92,the internal consistency reliability was alpha = 0.97 (33), and sensitivity and specificity 0.89 and 0.75, respectively (34).

### 2.3 Statistical analysis

The data were analyzed via SPPS© 27.0. The continuous variables are presented as the means ± standard deviations, whereas categorical descriptive data are presented as absolute values with percentages. Group comparisons of categorical data were performed by χ^2^ tests and continuous parametric and non-parametric data by the T-Test or Mann–Whitney U-test, respectively. To examine the correlation between inflammatory parameters and depression, we also used Pearson’s R correlation. In addition to the separate analyses performed for each psychological factor, we aimed to present the major assessed psychological outcomes together in a single model. Since our model includes many psychological factors that may be intercorrelated, we aimed to retain the most distinct variables. To exclude possible confounding factors and eliminate the least significant variables, we performed a stepwise backward conditional binomial logistic regression analysis. EA was the dependent outcome variable, and different psychological outcomes from questionnaires or interviews were independent variables. We reported odds ratios with 95% confidence intervals (CIs). If there was an outcome assessed in the interview and questionnaire, such as depressive disorders from the interview as a categorical variable and HADS-D score as a continuous variable, we preferred the continuous variable, as logistic function contributes to a more precise regression model outcome (35). We performed one additional analysis, replacing HADS-D with lifetime depression. The analysis was age- and gender-adjusted. In all statistical analyses, we considered a *p*-value ≤0.050 to be significant.

## 3 Results

Table 1 shows the study population description. The demographic data were obtained from all study participants and are presented in the Supplementary table.

**Table 1:** Study population description.

Early arthritis (*n* = 60)		Control group (*n* = 60)	
Early arthritis subgroups	n (%)	Control group subgroups	n (%)
RA	30 (50.0)	NIA	24 (40.0)
SpA	13 (21.7)	NA	36 (60.0)
UA	9 (15.0)		
MA	8 (13.3)		

RA, rheumatoid arthritis subgroup including seropositive, seronegative, and late-onset rheumatoid arthritis; SpA, spondylarthritis subgroup including psoriatic arthritis, axial spondylarthritis, and reactive arthritis; UA, undifferentiated arthritis; MA, miscellaneous arthritis including polymyalgia rheumatica, gout, sarcoidosis, connective tissue disease, and vasculitis. NIA, non-inflammatory arthralgia; NA, non-arthralgia.

### 3.1 Interview-based psychiatric disorders

**Figure 1:**
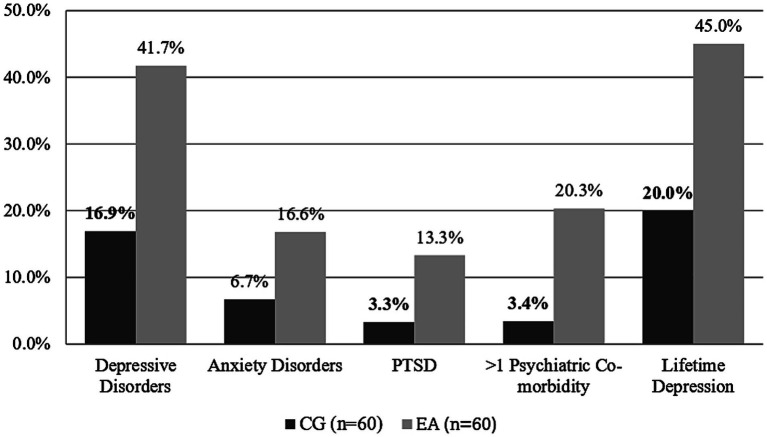
Prevalence of psychiatric co-morbidities in the semi-structured interview. Depressive disorders are a category for mild, moderate, and severe depressive episodes; recurrent depressive episodes; and adjustment disorders. Anxiety disorders include generalized anxiety, social phobia, agoraphobia, specific phobia, and PTSD. More than 1 psychiatric symptom indicated the coexistence of at least two psychiatric symptoms in the interview. Lifetime depression indicates at least one clinically relevant depressive episode in the lifetime. Bold figures indicate statistically significant differences between groups. CG, control group; EA, Early Arthritis; PTSD, Posttraumatic Distress Syndrome.

The psychiatric diagnoses from the interviews are presented in Figure 1. In the EA group, depressive disorders were significantly more prevalent than in the CG (*p* = 0.030). More than one psychiatric co-morbidity was significantly more common in EA patients (*p* = 0.036). When asked about a previous experience with MDD in the lifetime 27 (45%) of EA and 12 (20%) of HI answered positively (*p* = 0.003). The most common coexisting psychiatric co-morbidities were from the anxiety disorder spectrum. Overall, anxiety symptoms were not significantly more common in EA patients (*p* = 0.094). Six (66%) UA, seven (53.8%) SpA, three (37.5%) MA, and nine (30%) RA patients had a diagnosis of depression.

### 3.2 Psychological questionnaires

As shown in Table 2, there was a significant difference in mean HADS-D scores between EA and CG when compared by T-Test. We performed two additional analyses when we dichotomized the scores with cut-off points set at eight and 11. We found a statistically significant difference only with the lower threshold. When the four EA subgroups were compared, the SpA subgroup presented the highest levels of HADS symptoms, followed by the UA, MA, and RA subgroups.

**Table 2:** HADS and CTQ dimensions outcomes.

Psychological questionnaire results	Early arthritis (*n* = 60)	Control group (*n* = 60)	*p*-value
HADS depression			
Mean ± SD	5.4 ± 4.8	3.6 ± 3.3	**0.019**
Score ≥ 8, n (%)	18 (30.0)	9 (15.0)	**0.049**
Score ≥ 11, n (%)	8 (13.3)	3 (5.0)	0.114
HADS anxiety			
Mean ± SD	5.0 ± 3.8	4.6 ± 3.9	0.508
Score ≥ 8, n (%)	15 (25.0)	10 (16.7)	0.261
Score ≥ 11, n (%)	6 (10.0)	4 (6.7)	0.509
CTQ emotional abuse			
Mean ± SD	7.3 ± 3.5	7.6 ± 4.2	0.706
Score ≥ 13, n (%)	4 (6.7)	9 (15.0)	0.142
CTQ emotional neglect			
Mean ± SD	9.7 ± 4.8	8.4 ± 4.5	0.138
Score ≥ 15, n (%)	10 (16.7)	6 (10)	0.283
CTQ sexual abuse			
Mean ± SD	5.9 ± 2.7	5.1 ± 1.0	**0.042**
Score ≥ 8, n (%)	7 (11.7)	2 (3.3)	0.083
CTQ physical maltreatment			
Mean ± SD	6.1 ± 2.7	6.3 ± 3.0	0.655
Score ≥ 10, n (%)	5 (8.3)	6 (10.0)	0.752
CTQ physical neglect			
Mean ± SD	7.0 ± 2.8	6.4 ± 2.4	0.234
Score ≥ 10, n (%)	10 (16.7)	10 (11.7)	0.432

HADS, Hospital Anxiety and Depression Scale; SD, Standard Deviation; CTQ, Childhood Trauma Questionnaire. Bold *p*-values indicate statistical significance (*p*-value <0.05).

The total CTQ score for each dimension revealed that the mean CTQ score for childhood sexual abuse was significantly greater in the EA group than in the CG group (*p =* 0.042). The scores of the other CTQ dimensions were not significantly different between the EA and CG groups. Upon further examination, the NIA had notably high scores in all the CTQ dimensions. When we ran a separate analysis, we observed that 25% of the NIA subgroup experienced severe trauma in the dimensions of Emotional Abuse and Physical Neglect.

In our sample, 10 (8.3%) of all participants met the diagnostic criteria for current PTSD. Notably, eight (80%) of these patients had EA. This resulted in a significant statistical difference in comparison with the CG in the Chi-Square analysis (*p* = 0.049).

### 3.3 Clinical and laboratory parameters

The following clinical and laboratory parameters were obtained for the EA group. The mean disease duration was 4.8 ± 3.4 months. The mean levels of CRP and ESR were 30.5 ± 40.7 mg/L and 39.2 ± 28.7 mm/h, respectively. A total of 73.7 and 52.3% of EA patients exhibited CRP and ESR levels above the clinically relevant cut-off, respectively. ACPA and RF positivity were demonstrated in 29.8 and 33.3% of EA patients, respectively. The disease activity score 28 (DAS28CRP) mean score for the RA group in our sample was 3.5 ± 1.3. The mean pain visual analog scale (VAS) score in EA patients was 21.1 ± 25.2. EA patients reported a mean of 37.4 ± 42.3 min of morning stiffness daily. As EA patients were recruited at their first or second appointment, the majority were treatment-naive. At the time of study participation, 48 (80%) of EA and 60 (100%) of the NIA group received no glucocorticoid/csDMARD therapy.

### 3.4 Inflammatory parameters and correlation with depressive symptoms

We aimed to analyze the associations between inflammation and psychological outcomes in multiple examinations. Depression was the most prevalent psychiatric co-morbidity in our EA sample. Consequently, we performed analyses to observe the potential associations between depression and elevated inflammatory markers such as CRP, ESR and the anti-CCP, RF-IgM, and RF-IgA antibodies. With respect to psychiatric diagnoses, 22 (88%) of depressed EA patients showed elevated CRP levels as compared to 20 (62.5%) non-depressed patients (*p* = 0.030). There was a weak positive Pearson correlation (R = 0.29) between depression in the EA group and CRP. However, the CRP level did not correlate with the HADS-D sum score (*p* = 0.241, R = 0.13). The remaining inflammatory markers did not significantly correlate with depression. The ESR was elevated in 12 (63.2%) of the depressed patients and in 11 (44%) non-depressed patients, (*p* = 0.208). ACPA was elevated in 11 (42.3%) non-depressed and in three (14.3%) depressed EA patients (*p* = 0.101). Accordingly, RF-IgM/IgA positivity was almost evenly distributed across depressed and non-depressed patients, as was found in eight (32%) of depressed and 12 (34.3%) non-depressed patients (*p* = 0.853). The HADS-D score was positively correlated with VAS pain in the EA group (*p* = 0.001, R = 0.58). The DAS28CRP did not correlate with the HADS-D score within the RA group (*p* = 0.114, R = 0.3). Additionally, in our EA sample,there was no evidence for an association between depression and moderate/severe vitamin D deficiency, as a lack of vitamin D was present in nine (45.0%) of non-depressed and six (37.5%) of depressed patients (*p* = 0.861).

### 3.5 Logistic regression model outcomes

Logistic regression analysis revealed that HADS-D, CTQ emotional neglect, and CTQ sexual abuse were significantly associated with the presence of EA, with positive odds ratios, as shown in Table 3. An additional model was built when HADS-D scores were replaced with depressive episodes in the past; our model revealed HADS-D scores as strongly associated factors with the highest odds ratios (OR 4, confidence interval [CI] 1.55–10.33, *p* = 0.004). The CTQ emotional abuse score was a factor with a statistically significant *p-*value but a negative OR. Furthermore, to exclude other comorbid chronic diseases as confounders, we built seven different models with one comorbid disease added as an extra factor, while the rest of the model variables remained unchanged. The comorbid diseases we utilized were hypertension, coronary heart disease, hyper/hypothyroidism, diabetes, active tumors, renal insufficiency, and Crohn’s disease. These models resulted in outcomes comparable to those of the original model, suggesting that these diseases had little to no impact on either EA or psychiatric factors in our study population. We conducted additional analyses with different demographic parameters to include possible confounding factors. In supplementary analyses, educational level, weight, and marital status affect the model outcomes to some extent. The factors HADS depression, CTQ sexual abuse, and CTQ emotional abuse remained significant factors in the model. The CTQ emotional neglect was in these analyses not a significant independet factor. Smoking was identified as a confounder. When smoking was included as an independent variable, CTQ emotional abuse was the only factor that remained statistically significant (B = 0.85; *p* = 0.023). These analyses are provided in the Supplementary data.

**Table 3:** Binomial backward conditional regression model analysis adjusted for sex and age.

Variable	*p*-value	B	OR (95% CI)
HADS Depression	**0.025**	0.12	**1.12 (1.01–1.25)**
HADS Anxiety	0.238	−0.08	0.92 (0.80–1.06)
PTSD	0.286	−0.96	0.38 (0.66–2.22)
CTQ Emotional Abuse	**0.010**	−0.23	**0.79 (0.66–0.95)**
CTQ Emotional Neglect	**0.048**	0.12	**1.12 (1.01–1.26)**
CTQ Sexual Abuse	**0.040**	0.35	**1.42 (1.02–1.99)**
CTQ Physical Maltreatment	0.807	−0.02	0.95 (0.77–1.17)
CTQ Physical Neglect	0.500	0.07	1.06 (0.86–1.30)
Lifetime depression* Interview	**0.004**	1.38	**4.00 (1.55–10.33)**

Early Arthritis is an outcome variable in reference to the Control Group, and significant predictors are marked in bold. B, Beta, regression coefficient; OR, Odds Ratio; CI, Confidence Interval; SD, standard deviation; HADS, Hospital Anxiety and Depression Scale; PTSD, posttraumatic stress disorder; CTQ, Childhood Traumatic Questionnaire. Bold values indicate statistical significance (*p*-value < 0.05). *The lifetime depression variable was built in a separate model without HADS Depression.

## 4 Discussion

The debilitating nature of chronic inflammatory rheumatic diseases may affect mental health, as rheumatic patients often suffer from depression and anxiety symptoms (36–39). Rather than being only a co-morbidity, emerging studies suggest a bidirectional link between rheumatic and psychiatric diseases (40–42). To further investigate this topic, we compared the frequency of psychiatric co-morbidities and childhood trauma, as well as PTSD, in patients with EA at their first visit to controls without any manifestations of rheumatic disease. We explored whether these co-morbidities are potentially associated with EA. An additional goal was to determine the link between inflammatory markers and depression, one of the most prominent psychiatric conditions. Identifying the influential factors could be crucial for improving treatment outcomes in rheumatic diseases.

A semi-structuredinterview according to the DSM-V revealed that the frequency of depressive diagnoses was significantly higher in the EA group (42%) than in the control group (16%). Within the EA group, the UA group presented the highest rate of depression, followed by the SpA and RA groups. EA patients demonstrated more lifetime depressive episodes, which may imply that cumulative stressful events could lead to immune system malfunctions in the future. The mean HADS-D score was also significantly higher in the EA patients than in the CG. EA patients suffered more often from sexual abuse in childhood, as measured using the CTQ (OR 1.42, *p* = 0.040), and were four times more likely to experiencesymptoms of PTSD. Regression analysis suggests that current and lifetime depression, as well as sexual abuse and emotional neglect in childhood, were associated with the presence of EA. We discovered a positive correlation between depression in interviews and elevated CRP and VAS pain scores in EA patients.

Our results regarding depression are comparable to the few existing studies in EA patients and are in line with the results of the study of Bacconnier et al., who reported that 46.9% of EA patients described greater psychological distress at baseline. Interestingly, these authors reported a significantly lower rate of depression (25.8%) after a 3-yearfollow-up examination (43). These results suggest that symptoms of depression could be influenced by adequate anti-inflammatory therapy. Further studies concerning the link between different treatment responses and the psychological status of EA patients are needed. It could also be discussed whether the presence of depression or a history of depression could be a factor in the development of rheumatic disease. In the study of Fragoulis et al.,the HADS-D rates in EA were almost identical (8).

With respect to childhood trauma, there are considerable inconsistencies in the field; however, many studies suggest that emotional abuse and neglect are relevant factors in autoimmune diseases (44–46). We documented the high prevalence of childhood trauma in NIA patients. Approximately 25% of the NIA group experienced severe trauma in the CTQ emotional abuse and physical neglect dimensions. This finding may indicate a possible psychobiological element in NIA etiology. As early as 2018, Freier et al. reported a relatively high rate of depression and anxiety symptoms in NIA patients and discussed possible psychosomatic aspects (7). Alternatively, NIA might be an early form of future inflammatory joint disease. More prospective follow-up studies on this topic are needed.

The overall rate of PTSD in our study is slightly higher than in a study of Hellou et al., who reported a PTSD rate of 8.7% in patients with rheumatoid arthritis (47), which is comparable to the rate of 11.7% reported in the study of Mikulus et al. (48). Several studies, including one conducted on veterans of the Vietnam War, have highlighted the important relationship between PTSD and immune system dysregulation (21, 49). In addition, Lee et al. reported a higher incidence of RA in patients with PTSD symptoms (19).

Many studies are based on the so-called “cytokine theory” of depression, which suggests a possible link between inflammation and depression (41); however, clinical data regarding this topic are inconsistent (50). Compared with our results, Fragoulis et al. demonstrated a close association between increased CRP and the depression severity score in EA patients. This study also revealed no correlation between depressive symptoms and anti-CCP status in EA patients (51). This highlights the rather complex interactions between these two factors. A more obvious link is observed between depression and pain in EA, which has already been discussed by Freier et al. (7). Therefore, more studies regarding NIA patients are needed.

According to the regression model, lifetime depression was the factor most strongly associated with EA. Furthermore, emotional neglect and sexual abuse in childhood appear to influence EA manifestation according to our analysis. In contrast, we found a negative odds ratio for childhood emotional abuse, which was probably due to the fact that 25% of NIA had exposure to severe childhood emotional abuse. We must discourage the interpretation of the results of emotional abuse in childhood as a protective factor for early arthritis. To date, we cannot find comparative studies addressing this topic. In the supplementary analyses incorporating demographic variables in separate models, we observed that emotional neglect was no longer a significant factor. All other relevant variables remained significant. Moreover, smoking status, while not statistically significant, emerged as a largest confounder that influenced the outcomes of the model.

As most studies have examined established arthritis, one of the main strengths of our study is that the patients were in the very early stage of EA (symptom duration of 4.8 ± 3.4 months). In many cases, the exact diagnosis was still unknown at the time of the study interview. We conducted the semi-structured interview, in addition to the standardized psychological questionnaires, as a screening method for psychiatric diseases, which allowed us to explore not only the objective current state but also lifetime experiences that were not assessed with the subjective questionnaire. By integrating both screening techniques, we were able to differentiate between acute pain-related mood impairment and a manifest psychiatric diagnosis with greater precision. Expert consensus among psychiatrists and psychologists has utilized a variety of validated questionnaires for this purpose. Financial motivation did not lead to bias, as the participants were not compensated financially. The investigation into a so far neglected NIA group can be seen as a benefit of our study. However, this was also a limitation since this group had specific results. We classified the NIA group as part of the control group because there were no signs of inflammatory rheumatic disease in this group. However, we are aware that including this group might introduce bias into the healthy control group. While individuals in the NIA group did not meet diagnostic criteria for inflammatory joint disease, they nonetheless reported pain, which differentiates them from healthy individuals. The NIA group presented high rates of psychiatric comorbidities, which confirms the results of Freier et al. (7). Given that the study comprised 60 participants each in the EA and control groups, we restricted our analysis to descriptive statistics, as comparisons across groups of unequal size would not have been methodologically appropriate. For the same reason, we did not perform a supplementary sensitivity analysis using regression to compare the EA and NA groups. Supplementary analyses revealed that demographic factors had a confounding effect to a certain degree. Furthermore, interview bias cannot be completely ruled out. However, we tried to minimize this through additional supervision by a psychiatrist and structured interviews with standardized questions. An interviewer bias is less likely, as the interviewer was not aware of the exact diagnosis and laboratory results at the time of the interview. Further information regarding clinical and laboratory data was documented retrospectively, and the participants were then assigned to the NIA or the EA group. Another important limitation was the heterogeneity of the EA group. It consists of different inflammatory joint diseases, which can have varying clinical presentations and disease courses. Moreover, qualitative research is susceptible to attribution bias from the researcher as well as recall and social desirability bias from the participants’ side. The attribution bias might have been slightly reduced since, in many instances, the examiner was not aware of the exact diagnosis. We must also consider inclusion bias for all study groups, which can arise when participants consent to participate based on their past experiences or psychological issues. Given the time-intensive nature of our study with interviews and questionnaires, the study included relatively small sample sizes, which represents an additional limitation. This study identified a potential association between psychological factors and early arthritis. However, our design did not permit conclusion of causal relationships in either direction.

In conclusion, psychological factors such as current and lifetime depression, as well as sexual abuse and emotional neglect in childhood, are associated with the presence of EA in this investigation. The depression and PTSD rates in EA patients are high, which may influence their disease coping strategies. Clinicians should be aware of the high prevalence of depression in EA patients, especially patients with elevated CRP and high VAS pain scores. On the other hand, patients with NIA in our study had high scores for emotional abuse in childhood. Therefore, more research is needed to comprehend the possible psychobiological link between pain and childhood stress in this group. Thus, improvement in the psychological state could have a positive impact not only on coping with the disease but also on eventually alleviating pain symptoms in EA and NIA patients. More multimodal/psychological interventional research in EA is essential, as the long-term course of therapy, response to medication, and expression of co-morbidities are strongly related to the presence of depression (13, 43). According to our results, there is a massive psychological burden on EA patients. In addition to established psychiatric medications, non-pharmacological interventions, such as psychological counseling, psychotherapy, or participation in a self-help group, from the early stages of the disease may have a beneficial impact. It is necessary to conduct studies with both pharmacological and non-pharmacological interventions, as they may lessen the chronic nature of the disease and enhance the overall outcome of inflammatory joint diseases.

## 5 Conclusion

In our study, we discovered greater rates of depression, PTSD, and sexual abuse at the early stages of arthritis compared to healthy individuals. Moreover, we found an association between lifetime depression, childhood emotional neglect, and childhood sexual abuse with EA diagnosis. Given strong interaction between psychological factors and early arthritis, early psychological screening and intervention might potentially enhancemental health outcomes while alleviating early arthritis symptoms.

## Data Availability

The raw data supporting the conclusions of this article will be made available by the authors, without undue reservation.

## Glossary

EA: early arthritis
CG: control group
NIA: non-inflammatory arthralgia
NA: non- arthralgia
RA: rheumatoid arthritis
SpA: spondylarthritis
UA: undifferentiated arthritis
MA: miscellaneous arthritis diagnosis
HADS-D: Hospital Anxiety and Depression Scale – Depression
HADS-A: Hospital Anxiety and Depression Scale – Anxiety
CTQ: Childhood Trauma Questionnaire
EA: emotional abuse
PA: physical abuse
SA: sexual abuse
EN: emotional neglect
PN: physical neglect
PDS: Posttraumatic Diagnostic Scale
PTSD: post-traumatic stress disorder
SD: standard deviation
B: Beta, regression coefficient
OR: odds ratio
95% CI: 95% confidence interval
R: Pearson correlation
CRP: C-reactive protein
ESR: erythrocyte sedimentation rate
RF-IgA: rheumatoid factor IgA
RF-IgM: rheumatoid factor IgM
ACPA: Anti- citrullinated protein antibody
DAS28-CRP: Disease Activity Score 28 with CRP
VAS: visual analog scale
EULAR/ACR: European League Against Rheumatism /American College of Rheumatology
HPA Axis: Hypothalamus-Pituitary–Adrenal Axis
IL-6: Interleukin 6
TNF-*α*: Tumor Necrosis Factor-alpha
BMI: body mass index
DSM-V: Diagnostic and Statistical Manual of Mental Disorders, Fifth Edition
