# Supplementary material for: Associations between childhood trauma, depression, anxiety disorders and early arthritis presence
Source: Front Med (Lausanne). 2025 Aug 5;12:1582075. doi: 10.3389/fmed.2025.1582075 (PMC12361254; doi:10.3389/fmed.2025.1582075)
Supplement: Supplementary file 1 [file Table_1.docx]

Supplementary file

Table 1

Demographic Characteristics

|  | **Early**  **Arthritis**  **(n=60)** | **Control**  **Group**  **(n=60)** | ***p*-Value** |
| --- | --- | --- | --- |
| **Sex** |  |  |  |
| Female, n (%) | 35 (58.3) | 35 (58.3) | 1 |
| **Age** (mean ± SD) | 49.6 + 15.9 | 45.3 ± 16.0 | 0.14 |
| **BMI** (mean ± SD) | 25.9 +7.2 | 24.9± 4.3 | 0.40 |
| **Marital Status, n (%)** |  |  |  |
| - Single | 13 (22.0) | 17 (28.8) | 0.59 |
| - Married | 33 (55.9) | 38 (64.4) |  |
| - Divorced/Widowed | 13 (22.0) | 4 (6.8) |  |
| **Smoking Status, n (%)** |  |  |  |
| - Active Smokers | 21 (36.8) | 23 (31.2) | 0.83 |
| - Ex-Smokers | 11 (19.3) | 15 (20.8) |  |
| - Non-Smokers | 25 (43.9) | 25 (47.9) |  |
| **Education, n (%)** |  |  |  |
| - Elementary School or less | 6 (10.0) | 4 (6.7) | 0.09 |
| - High School or less* | 17 (28.3) | 7 (11.7) |  |
| - German Abitur (High School Graduation) ** | 12 (20.0) | 17 (28.3) |  |
| - University or university equivalent | 22 (36.7) | 31 (51.7) |  |
| **Comorbid Factors, n (%)** |  |  |  |
| - Two or more comorbid diseases | 10 (16.7) | 3 (5) | 0.08 |
| SD = standard deviation, BMI = Body Mass Index, *High school or less= Mittlere Reife, Realschuleabschluss, Polytechnische Oberschule in Germany, **High School Graduation = Abitur in Germany. Comorbid factors: hypertension, coronary heart disease, hyper-/hypothyroidism, diabetes mellitus, active tumor, renal insufficiency, and Morbus Crohn's disease. | | | |
