# Supplementary material for: Associations between childhood trauma, depression, anxiety disorders and early arthritis presence
Source: Front Med (Lausanne). 2025 Aug 5;12:1582075. doi: 10.3389/fmed.2025.1582075 (PMC12361254; doi:10.3389/fmed.2025.1582075)
Supplement: Supplementary file 2 [file Table_2.docx]

Supplementary table 2. Inflammation markers and rheumatologic antibodies in EA

| **Parameter** | **n** | **Mean ± SD** | **Median** | **Minimum** | **Maximum** | |
| --- | --- | --- | --- | --- | --- | --- |
| CRP (mg/L) | 57 | 30.47 ± 40.69 | 13.50 | 0.40 | 177.70 |  |
| ESR (1h, mm) | 45 | 39.20 ± 28.70 | 6.30 | 6.00 | 100.00 |  |
| ACPA (U/mL) | 46 | 136.21 ± 280.89 | 2.10 | 0.00 | 1000.00 |  |
| RF-IgM (U/mL) | 55 | 36.62 ± 79.48 | 4.20 | 0.00 | 500.00 |  |
| RF-IgA (U/mL) | 55 | 32.36 ± 78.57 | 7.90 | 0.00 | 500.00 |  |
