# Supplementary material for: Associations between childhood trauma, depression, anxiety disorders and early arthritis presence
Source: Front Med (Lausanne). 2025 Aug 5;12:1582075. doi: 10.3389/fmed.2025.1582075 (PMC12361254; doi:10.3389/fmed.2025.1582075)
Supplement: Supplementary file 3 [file Table_3.docx]

Supplementary Table 3. Binomial backwards conditional regression model analysis final outcome, adjusted for sex and age. Early Arthritis is an outcome variable in reference to the Control Group; significant predictors are marked bold. In each model, the original independent variables (HADS-D, HADS-A, CTQ Emotional Abuse, CTQ Emotional Neglect, CTQ Physical Abuse, CTQ Physical Neglect, CTQ Sexual Abuse, and PTSD) were included, with the addition of one different independent variable (Weight, Education, Marital Status, and Smoking Status) tested separately in each analysis.

| Variable 1. Weight |  |  |  |  | |
| --- | --- | --- | --- | --- | --- |
|  | **p-Value** | **B** | **OR** | | **(95% CI)** |
| HADS Depression | **0.031** | 0.12 | **1.12** | | (1.00 – 1.25) |
| CTQ Emotional Abuse | **0.011** | –0.23 | **0.79** | | (0.66 – 0.95) |
| CTQ Sexual Abuse | **0.026** | 0.42 | **1.53** | | (1.05 – 2.21) |
| CTQ Emotional Neglect | 0.087 | 0.10 | 1.10 | | (0.98 – 1.25) |
| Weight | 0.705 | 0.01 | 1.00 | | (0.98 – 1.02) |

Variable 2. Education

| HADS Depression | 0.022 | 0.12 | 1.13 | (1.02 – 1.26) |
| --- | --- | --- | --- | --- |
| CTQ Emotional Abuse | **0.018** | –0.21 | **0.81** | (0.67 – 0.96) |
| CTQ Sexual Abuse | **0.049** | 0.35 | **1.42** | (1.00 – 2.00) |
| CTQ Emotional Neglect | 0.094 | 0.10 | 1.11 | (0.98 – 1.24) |

Variable 3. Marital Status

| HADS Depression | 0.020 | 0.13 | 1.14 | (1.02 – 1.27) |
| --- | --- | --- | --- | --- |
| CTQ Emotional Abuse | **0.016** | –0.22 | **0.80** | (0.67 – 0.96) |
| CTQ Sexual Abuse | **0.048** | 0.35 | **1.42** | (1.00 – 2.02) |
| CTQ Emotional Neglect | 0.101 | 0.10 | 1.10 | (0.98 – 1.24) |

Variable 4. Smoking status

| HADS Depression | 0.072 | 0.10 | 1.11 | (0.99 – 1.24) |
| --- | --- | --- | --- | --- |
| CTQ Emotional Abuse | **0.028** | –0.15 | **0.86** | (0.74 – 0.98) |
| CTQ Sexual Abuse | 0.061 | 0.31 | 1.37 | (0.99 – 1.90) |

Abbreviations: B= Beta, regression coefficient; OR = Odds Ratio; CI = Confidence Interval; SD = standard deviation; HADS = Hospital Anxiety and Depression Scale; PTSD = posttraumatic stress disorder; CTQ = Childhood Traumatic Questionnaire. Bold values indicate statistical significance (*p*-value < 0.05).
